# Neurotrophic Factors in Health and Disease

**DOI:** 10.3390/cells12010047

**Published:** 2022-12-22

**Authors:** Cinzia Severini

**Affiliations:** Institute of Biochemistry and Cell Biology (IBBC), National Research Council, Viale del Policlinico, 155-00161 Rome, Italy; cinzia.severini@cnr.it

Neurotrophic factors, including neurotrophins and neuropeptides, are secreted proteins that regulate the survival, development, and physiological functions of neurons in both the central and peripheral nervous systems. These factors are not only essential for normal neuronal activity but may also play a role in neurodegeneration following trauma, ischemia, and neuroinflammatory reactions. A deficiency of neurotrophic factors or the deregulation of their cognate receptors is causally associated with the onset and progression of neurodegenerative diseases, i.e., Alzheimer’s disease (AD), Huntington’s disease (HD), amyotrophic lateral sclerosis (ALS), and Parkinson’s disease (PD). In addition to neurodegenerative diseases, neurotrophic factors are also involved in the modulation of cellular processes in the peripheral nervous system and participate in disorders in various tissues, such as muscle, and the cardiovascular system.

This Special Issue of *Cells* summarizes the current knowledge regarding the roles of different neurotrophic factors in health and various diseases, focusing on the complex cell biology and pharmacology of these factors on the cellular, molecular, and clinical levels.

The work of Sebastiano Cavallaro [1] and his collaborators, who analyzed the transcriptional profiles of cerebellar granule cells during the early commitment phase of apoptosis or their rescue by three different neurotrophic factors (Pacap, Igf1, and substance P), identified a core set of genes that show opposite expression trends at the interface between apoptosis and survival, providing a systems-biology-based perspective on the earliest molecular events controlling neuronal fate decisions.

Interestingly, the genes in the apoptosis/survival core set are significantly correlated with neurological, psychiatric, and oncological diseases and encode therapeutic targets whose modulation may exert anti-apoptotic or pro-survival effects.

Among the neurotrophic factors, nerve growth factor (NGF), which regulates neuron survival and differentiation, has been extensively studied for its neuroprotective effects, especially in AD, in which NGF plays a central role [2].

In our studies [3], we investigated the role of NGF in rat primary cortical cultures and hippocampal slices of mice and AD transgenic mice to whom NGF was administered intranasally. We demonstrated the involvement of bradykinin (BK) receptor 2 (B2R) in the mechanism of action of NGF and synaptic plasticity and identified the microglial cells as the target of this modulation. Thus, we confirmed the findings of previous studies, indicating that BK and its receptors are important factors in AD human pathology and animal models [4].

In the brain, NGF regulates not only neuron survival and differentiation but also glial and microglial functions and neuroinflammation. Antonino Cattaneo and his group [5] found that NGF controls oligodendrogenesis by modulating the levels of miR-219a-5p in an NGF deprivation mouse model, AD11 mice. They demonstrated increased levels of miR-219a-5p in the AD11 hippocampus and cortex, along with increased myelination. The treatment of AD11 neurospheres with NGF inhibits the upregulation of miR-219a-5p and, consequently, oligodendrocyte differentiation and myelination. This suggests the possibility of a new target for human demyelinating diseases, such as multiple sclerosis.

On the peripheral level, NGF exerts pleiotropic effects on various cell types and tissues, including skeletal muscle. Marco Segatto and coworkers [6] examined the involvement of the ProNGF/p75NTR axis in mouse skeletal muscle both in vitro and in vivo using mdx mice (a model of Duchenne muscular dystrophy). The author showed that the modulation of the proNGF/p75NTR pathway, which controls fiber type determination, could provide an innovative therapeutic approach to counteract muscle diseases.

Another member of the neurotrophin family, brain-derived neurotrophic factor (BDNF), has been shown to be involved in numerous neurodegenerative and neuropsychiatric disorders by regulating axonal guidance, promoting synaptic plasticity and neurite growth, and facilitating long-term potentiation [7].

In the review conducted by Carmel Matrone et al. [8], the functional effect of musical stimulation on BDNF expression is analyzed in both animal models and humans. Musical stimulation is compared to motor activity and is able to consistently increase the BDNF levels, promoting neurogenesis and synaptic plasticity and improving cognitive performance. The authors suggest that music, along with motor exercise, may be a novel strategy for maintaining or restoring cognitive abilities.

The paper of Patrizia Campolongo and her group [9], which examined the response to social defeat stress (SDS) in rats, indicated that animals exposed to SDS in early adolescence and then to a single episode of prolonged stress (SPS) in adulthood exhibited resistance to the development of changes in emotionality, arousal, and spatial memory. This demonstrates how social stress in early adolescence can influence the ability to cope with a second challenge later in life by modulating BDNF expression in the hippocampus and the plasma corticosterone levels.

In addition, BDNF is involved in the homeostasis of the cardiovascular system. The role played by BDNF Val66Met polymorphism in myocardial infarction was investigated by Stella Barbieri and coworkers [10]. Using mice carrying the human BDNF Val66Met polymorphism, the authors found that such BDNF polymorphism predisposes individuals to adverse cardiac remodeling after myocardial infarction and directs the macrophages towards a pro-inflammatory M1-like phenotype with a higher migratory capacity, suggesting the identification of a novel target in cardiovascular disease.

Another neurotrophic factor studied in this Special Issue is epidermal growth factor (EGF) and its related tyrosine kinase receptor (EGFR). Cecilia Bucci and collaborators [11] investigated EGFR degradation in a pathogenic phenotype associated with a novel RAB7A mutation in the rare Charcot–Marie–Tooth type 2B (CMT2B), a disease characterized by predominant sensory deficits, a high incidence of ulceration, and variable motor deficits, in a clinical study. The authors pointed to the reduced degradation of EGFR as a key factor in pathogenicity in CMT2B patients carrying this mutation.

Last but not least, the involvement of vascular endothelial growth factor (VEGF) in depression was investigated by Daniela Pollak and coworkers using a model of maternal immune activation in rats (MIA). The offspring developed depression-like behavior associated with the decreased expression of VEGF receptor 2 (VEGFR2) in the hippocampus [12]. In adulthood, intracerebroventricular VEGF infusion improved the behavioral deficits in the MIA male offspring, suggesting that VEGF treatment has therapeutic potential for the MIA model of depression.

Overall, the papers published in this Special Issue offer new insights into this scientific field and illustrate the key roles that trophic support can play, both from a physiological point of view and in the development of pathologies that are not limited to the central nervous system but also relate to various organs and tissues, stimulating further research.
